# Measles Surveillance in Tuscany (Italy), 2019–2024: A Six-Year Epidemiological Analysis

**DOI:** 10.3390/vaccines14070558

**Published:** 2026-06-25

**Authors:** Manuela Chiavarini, Rossella Romano, Andrea Guida, Elena Morelli, Camillo Di Nizio, Teresa Vladina Picchi, Giulia Napoli, Roberta Murolo, Vittorio Filieri, Barbara Rita Porchia, Daniela Senatore, Giovanna Bianco, Paolo Bonanni, Sara Boccalini, Angela Bechini

**Affiliations:** 1 Department of Health Sciences, University of Florence, 50134 Florence, Italy; 2 School of Specialization in Hygiene and Preventive Medicine, Department of Health Sciences, University of Florence, 50134 Florence, Italy; 3 Azienda USL Toscana Centro, 50122 Florence, Italy; 4 Regione Toscana, Direzione Sanità, Welfare e Coesione Sociale, 50127 Florence, Italy

**Keywords:** Morbillivirus Infections, measles, outbreak, epidemiology, immunization, vaccination, public health preparedness, epidemic, children, adults

## Abstract

Background: Despite sustained public-health efforts, measles continues to re-emerge in Europe. According to the ECDC, 1045 measles cases were reported in Italy in 2024. We describe epidemiological trends and characteristics of measles cases in the Tuscany Region from 2019 to 2024. Methods: We conducted a population-based retrospective study using cases reported through the national surveillance system (PREMAL). Incidence rates were calculated using demographic data from the Italian National Institute of Statistics (ISTAT). Cases were stratified by year, sex, age group, vaccination status (2024), and hospital admission; temporal, demographic, and clinical trends were analysed. Results: From 2019 to 2024, 204 cases were reported, corresponding to a mean annual incidence of 0.93 per 100,000 population (95% CI: 0.80–1.07) and a cumulative incidence of 5.58 per 100,000 (95% CI: 4.78–6.31). Females accounted for 63.2% of cases (*n* = 129). After a peak in 2019 (*n* = 116), with an incidence of 3.13 per 100,000 (95% CI: 2.56–3.71), cases sharply declined during 2020–2023, followed by a resurgence in 2024 (*n* = 75), with an incidence of 2.05 per 100,000 (95% CI: 1.59–2.51). Children aged 0–4 years represented 7.4% of cases but had the highest age-specific incidence (12.19 per 100,000). Adults aged 25–64 years accounted for 70.1% of all cases, indicating the greatest absolute burden. Incidence was higher among individuals aged 25–44 years (11.56 per 100,000) than among those aged 45–64 years (4.30 per 100,000). Overall, 41.7% of cases required hospitalization. In 2024, most cases occurred in unvaccinated individuals (*n* = 56), while vaccination status was unknown for five cases. Conclusions: The 2024 measles resurgence in Tuscany mainly affected unvaccinated adults. These findings highlight persistent immunity gaps among adults, suggesting that protection and prevention measures are also needed in the population (0–4 y).

## 1. Introduction

Measles remains a public health problem worldwide and across the European Union and the European Economic Area (EU/EEA), including Italy, which has reported among the highest numbers of cases in recent years. According to data released by the Istituto Superiore di Sanità (ISS), 1045 cases of measles were reported in 2024 in Italy [1]. Consistently, the European Centre for Disease Prevention and Control (ECDC) reported 1097 cases in Italy between 1 February 2024 and 31 January 2025 [2]. Across the EU/EEA, 35,212 measles cases were reported in 2024, representing a tenfold increase compared with 2023 and indicating a marked resurgence of cases after years of decline [2]. Globally, measles continues to pose a substantial health burden, with an estimated 10.3 million cases and more than 100,000 deaths in 2023, a 20% increase compared with 2022 [3].

Measles is a highly contagious viral infection that is transmitted through the air; it can affect people of all ages and spread rapidly within a susceptible population, for example, those with inadequate vaccination coverage [4]. Complications may involve multiple organ systems and include otitis media, diarrhoea, pneumonia—the leading cause of death in affected children—and encephalitis, with more severe outcomes in vulnerable groups. Achieving and maintaining high vaccination coverage is essential to ensure herd immunity, reduce disease incidence, and prevent new outbreaks [5]. The World Health Organization (WHO) recommends maintaining at least 95% coverage with two doses of a measles-containing vaccine (MCV) in all districts as a prerequisite for measles elimination, together with the implementation of highly sensitive surveillance systems capable of rapidly detecting and investigating suspected cases. New strategies for implementing measles vaccination processes have recently been adopted, such as the WHO’s Immunization Agenda 2030 (IA2030) [6]. IA2030, approved during the 73rd World Health Assembly (2020), identifies measles as a key indicator of health system performance and progress towards sustainable development goals and of ensuring health and well-being for all ages.

In addition, the National Plan for the Elimination of Measles and Rubella, introduced in Italy in 2003, aims to interrupt endemic transmission through strengthened vaccination programmes, catch-up campaigns for susceptible groups, and a surveillance system [7,8]. In Italy, the National Vaccination Prevention Plan (Piano Nazionale di Prevenzione Vaccinale—PNPV) 2023–2025 recommends two doses of measles-containing vaccine: the first at 12 months of age and a booster dose at 5 years [7,8]. Since 2017, measles vaccination has been mandatory for school enrolment, contributing to increased coverage; however, the 95% target for elimination has not yet been achieved [9].

In Italy, integrated surveillance of measles and rubella is coordinated by the Department of Infectious Diseases of the ISS with support from the National Reference Laboratory for Measles and Rubella [10,11]. The system is designed to ensure the timely detection and laboratory confirmation of suspected cases, early identification of outbreaks, and prompt public health response, including case and contact management. It also provides continuous monitoring of transmission patterns, particularly in settings with suboptimal vaccination coverage or importation-related risk, enabling the identification of vulnerable population groups and the implementation of targeted control measures. In addition, surveillance data are essential to track disease incidence and document progress toward national and international measles elimination targets [12].

Despite the availability of national-level data, regional analyses remain limited. This study aims to analyse measles cases in Tuscany for the period 2019–2024, describing epidemiological trends, demographic and clinical characteristics, and population groups at increased risk, in order to provide useful information for improving prevention and control strategies at the regional level.

## 2. Materials and Methods

### 2.1. Study Setting and Population

This population-based retrospective surveillance study was conducted in the Tuscany Region, Italy. As of 31 January 2025, the resident population was 3,660,530, distributed across three Local Health Units (LHUs): Tuscany Centre (1,605,304 inhabitants), Tuscany North-West (1,244,707), and Tuscany South-East (807,705). The Tuscany Centre LHU includes the provinces of Florence, Prato, and Pistoia; North-West includes Lucca, Pisa, Massa Carrara, and Livorno; and South-East includes Arezzo, Siena, and Grosseto.

### 2.2. Data Source, Case Definitions, and Surveillance System

Measles cases notified between 2019 and 2024 were extracted from the national PREMAL surveillance system, a passive reporting platform for notifiable diseases based on routine notifications from healthcare providers and LHUs. The system ensures nationwide coverage and supports the long-term monitoring of temporal and demographic trends, outbreak detection, and measles elimination activities by collecting epidemiological, clinical, laboratory, and vaccination data as part of routine public health practice. Case definitions and classifications were based on the national surveillance protocol and were aligned with WHO and European Union case definitions [9,10,13]. A suspected case was defined as any person with fever and maculopapular rash plus cough, coryza, or conjunctivitis or any clinically suspected measles case. A laboratory-confirmed case was a suspected case with laboratory evidence of recent measles virus infection. An epidemiologically linked case was a suspected case without laboratory confirmation but linked to a confirmed case through epidemiological investigation; cases not meeting these criteria were classified as discarded. Case origin was classified according to WHO criteria as imported, import-related, endemic (locally acquired), or of unknown source. Imported cases were infections acquired outside Italy during the incubation period, while import-related cases were linked to an imported case; endemic cases reflected local transmission, and cases with insufficient information were classified as unknown. A measles outbreak was defined as ≥2 epidemiologically linked confirmed cases within a defined temporal and geographical setting, according to national surveillance guidelines [10,11]. Where available, molecular surveillance data were used to identify circulating measles virus genotypes and support transmission analysis, although genotype information was not systematically available and was therefore not included in the analysis. Variables extracted from PREMAL included year of notification, age group, sex, Local Health Unit of residence, and hospitalization status; month of notification was not consistently available across the study period. Since 2024, standardized reporting forms have included additional variables such as disease severity and healthcare worker status. However, laboratory confirmation and vaccination status were not consistently available throughout the study period [14]. Vaccination data were available only for 2024 cases through the measles–rubella surveillance system coordinated by the ISS [10,11].

### 2.3. Statistical Analysis

Descriptive analyses were used to assess the distribution of cases by year, sex, age group, and LHU of residence. Hospitalizations were analysed as both absolute numbers and proportions of reported cases. Incidence and hospitalization rates per 100,000 residents were calculated using annual ISTAT population denominators. Period incidence rates over the study period were calculated using the average resident population as the denominator [15]. For each rate, 95% confidence intervals (CIs) were calculated to quantify statistical uncertainty and to assess estimate precision. For small numbers of events (≤5), exact Poisson confidence intervals were applied. All analyses were performed using Microsoft Excel (2019 version; Microsoft Corporation, Redmond, WA, USA).

## 3. Results

**Figure 1:**
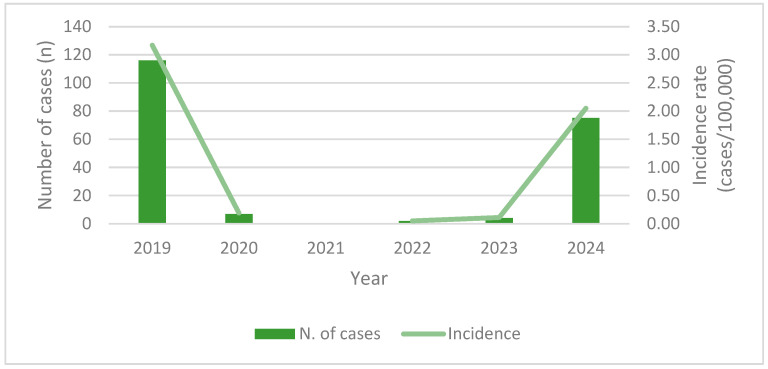
Annual measles cases and incidence rates; Tuscany Region, 2019–2024.

Between 2019 and 2024, a total of 204 measles cases were reported in Tuscany, corresponding to a mean annual incidence rate of 0.93 per 100,000 population (95% CI: 0.80–1.07). The highest number of cases was observed in 2019 (*n* = 116 notifications; incidence of 3.13 per 100,000 (95% CI: 2.56–3.71). Between 2019 and 2024, measles incidence showed marked temporal variation, with the highest rates observed in 2019 and a resurgence in 2024 (*n* = 75), an incidence of 2.05 per 100,000 (95% CI: 1.59–2.51), after a period of very low or no reported incidence. The 2024 notifications accounted for 36.8% of all cases reported during the study period, while the 2019 outbreak represented 56.9% of the cumulative case burden. Together, these two years accounted for 93.6% of all reported measles cases between 2019 and 2024. The cumulative incidence rate was 5.58 cases per 100,000 inhabitants (95% CI: 4.78–6.31) with substantial variation between years (Supplementary Table S1; Figure 1).

Female cases consistently accounted for a higher proportion across the study period (*n* = 129; 63.2%). The female-to-male ratio was 1.7:1 overall, ranging from 1.6:1 in 2019 to 2.0:1 in 2024. Female incidence rates matched those of males in two years and exceeded them in all remaining years with reported cases, often by a substantial margin.

**Figure 2:**
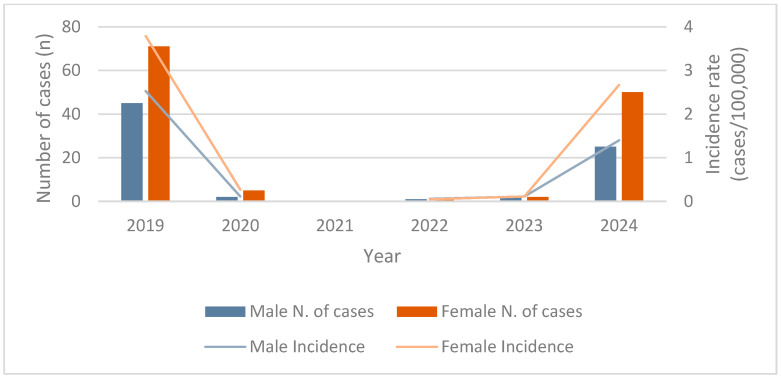
Annual measles cases and incidence rates by sex in Tuscany, 2019–2024.

Over the 2019–2024 period, the mean annual incidence rate was 0.70 per 100,000 (95% CI: 0.54–0.86) among males and 1.13 (95% CI: 0.94–1.33) among females (Supplementary Table S1; Figure 2).

**Figure 3:**
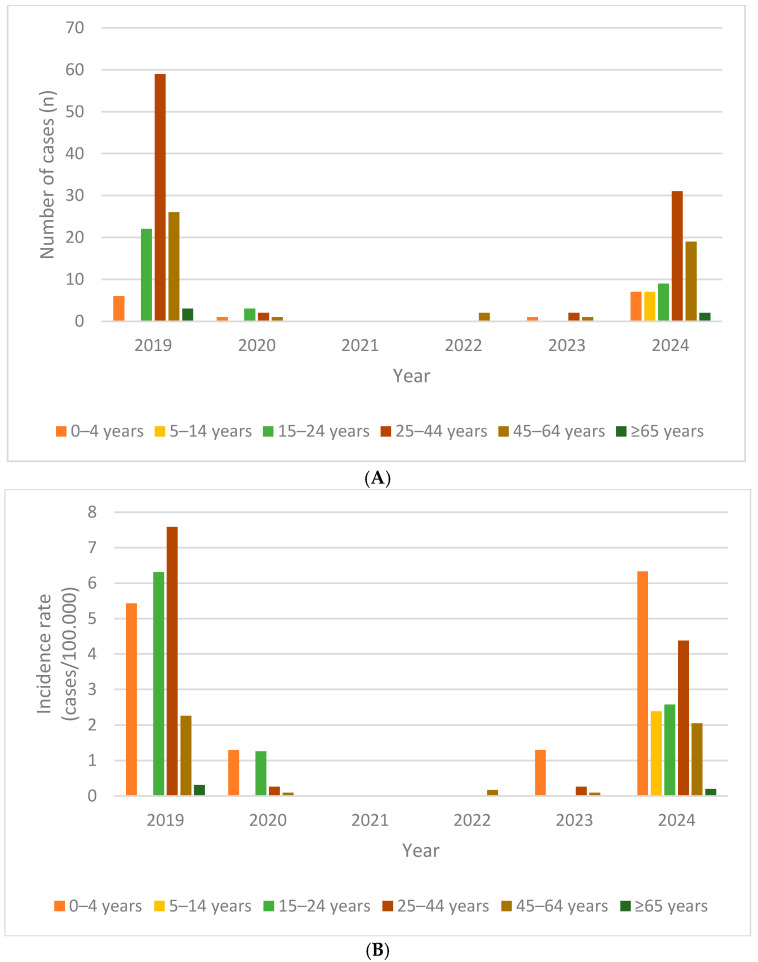
Annual measles cases (**A**) and incidence rates (**B**) by age group in Tuscany, 2019–2024.

Adults aged 25–64 years represented the most affected age group throughout the study period, accounting for 70.1% of all reported cases (*n* = 143), ranging from 66.7% (*n* = 50) in 2024 to 100% in 2022 (*n* = 2) (Supplementary Table S1, Figure 3A). Within this category, individuals aged 25–44 years accounted for 46.1% of all reported cases (*n* = 94), whereas those aged 45–64 years represented 24.0% (*n* = 49). Together, these two age groups accounted for nearly three-quarters of all reported cases.

Incidence was highest among young children (0–4 years), adolescents and young adults (15–24 years), and individuals aged 25–44 years, who consistently exhibited the highest incidence rates across the study period. Over the entire period, the highest cumulative incidence rates were observed among children aged 0–4 years (12.19 per 100,000 population), adults aged 25–44 years (11.56 per 100,000), and individuals aged 15–24 years (10.17 per 100,000). In contrast, incidence remained consistently low among individuals aged 5–14 years and older adults (≥65 years), with cumulative incidence below 1 case per 100,000 in the latter group (Supplementary Table S1, Figure 3B).

In 2019, the highest incidence was recorded among adults aged 25–44 years (6.89 per 100,000), who also accounted for the highest number of cases (*n* = 59). By 2024, the peak incidence had shifted to children aged 0–4 years (6.19), although the highest number of cases remained within the 25–44 years age group (*n* = 31).

**Figure 4:**
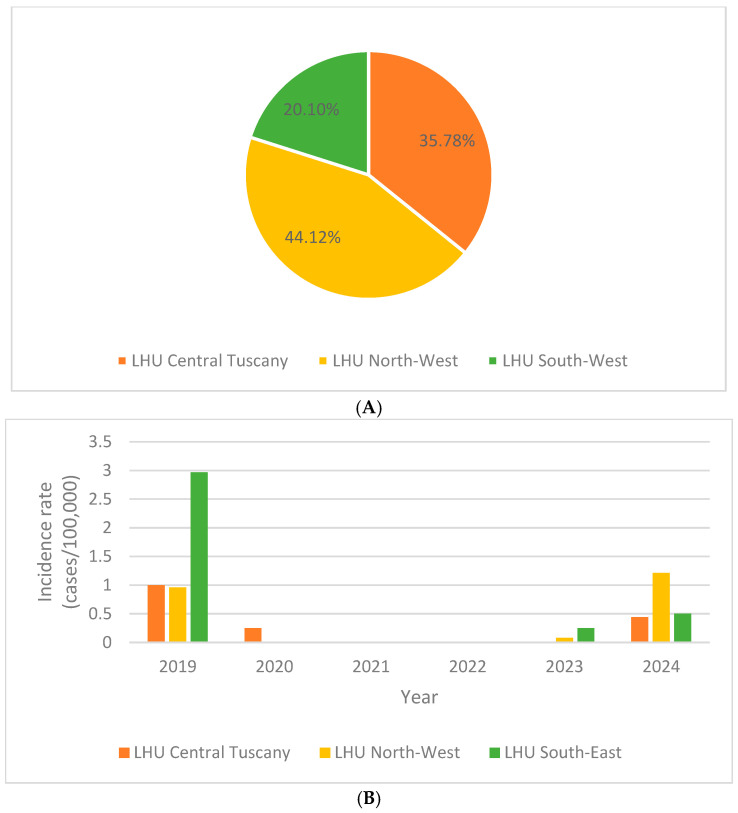
Distribution of measles cases (%) by Local Health Unit (LHU) (**A**) and annual incidence rates by LHU (**B**) in the Tuscany Region, 2019–2024.

The Tuscany North-West LHU reported the majority of cases, both in 2024 (*n* = 49, 65.3%) and over the entire 2019–2024 period (*n* = 90, 44.1%) (Supplementary Table S1, Figure 4). Across the study period, 35.8% of cases were reported in the Tuscany Centre LHU (*n* = 73), 44.1% in the Tuscany North-West LHU (*n* = 90), and 20.1% in the Tuscany South-East LHU (*n* = 41).

**Figure 5:**
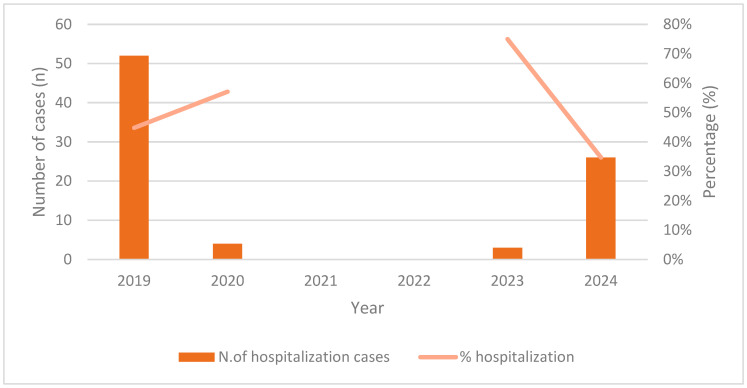
Annual measles-related hospitalizations and hospitalization percentage among notified cases in Tuscany, 2019–2024.

Overall, 85 cases required hospitalization, representing 41.7% of all reported cases. The proportion of hospitalized cases varied across years, ranging from 34.7% in 2024 to 75.0% in 2022; however, the latter estimate was based on a limited number of notified cases. In absolute terms, most hospitalizations occurred in 2019 (*n* = 52) and 2024 (*n* = 26), together accounting for 91.8% of all measles-related hospital admissions recorded during the study period. The mean annual hospitalization rate was 2.32 per 100,000 inhabitants per year (95% CI: 1.8–2.8) (Supplementary Table S1). Hospitalization rates by LHU reflected the temporal trend observed for measles incidence and are presented in Supplementary Table S1. The highest incidence rates were observed in 2019 (3.13/100,000; 95% CI: 2.56–3.71) and 2024 (2.05/100,000; 95% CI: 1.59–2.51). The distribution of hospitalized cases differed between these two years with respect to both age group and sex (Figure 5).

According to the ISS surveillance data for the Tuscany region, the vast majority of measles cases in 2024 occurred among the Italian-born population; individuals born abroad accounted for only 10% (*n* = 9) of the total reported cases. Among the 75 cases reported in 2024, 69.3% occurred among adults aged ≥25 years (*n* = 52), whereas children aged 0–4 years accounted for 9.3% (*n* = 7); the median age was 32 years, ranging from 0 to 73 years. Females represented nearly two-thirds of all reported cases (66.7%, *n* = 50).

Healthcare workers accounted for 5.3% of the cases (*n* = 4). Vaccination status was available for most cases notified in Tuscany: 70.6% were unvaccinated (*n* = 53), 21.3% had received at least one dose of measles vaccine (*n* = 16), and 6.6% had unknown vaccination status (*n* = 5). Among those vaccinated, the average time since last vaccination was 10 years (range: 0–31 years). Nearly all cases presented with rash and fever (the latter in 93.3%, *n* = 70), followed by cough (72.0%, *n* = 54), conjunctivitis (54.6%, *n* = 41), rhinitis (53.3%, *n* = 40), arthralgia (36.0%, *n* = 27), and lymphadenopathy (29.3%, *n* = 22). With regard to geographical distribution, all cases in the Tuscany Centre LHU were reported in the Province of Florence. In the Tuscany North-West LHU, the majority of cases were reported in the Province of Pisa (*n* = 39), accounting for 79.6% of all cases in the Northern LHU, while in the Tuscany South-East LHU, most cases occurred in the Province of Siena (*n* = 7), representing 87.5% of all cases in the area. Overall, these provinces together accounted for more than 85% of all notified cases. At the provincial level, Pisa accounted for 52.0% of all cases reported in Tuscany in 2024 (*n* = 39), followed by Florence (24.0%, *n* = 18) and Siena (9.3%, *n* = 7). During the surveillance period, no deaths due to measles were reported.

## 4. Discussion

The epidemiology of measles in Tuscany during the study period reflects the broader re-emergence of measles observed in Italy and internationally, confirming the persistence of immunity gaps in populations where elimination targets have not yet been sustainably achieved [16,17,18,19]. Two epidemic phases clearly emerged in Tuscany: the pre-pandemic outbreak in 2019 and the resurgence in 2024. Although separated by a period of minimal circulation, both waves likely originated from the same structural determinant: the accumulation of susceptible individuals due to suboptimal vaccination uptake over time [20,21].

The 2019 epidemic in Tuscany was characterized mainly by transmission among adolescents and young adults, consistent with patterns observed in previous Italian outbreaks [22]. This distribution suggests the persistence of susceptible cohorts born between the late 1970s and early 1990s, who may not have received one or both doses of measles-containing vaccine. These cohorts have now reached working and reproductive age, representing epidemiologically relevant reservoirs due to their high social connectivity and mobility.

In contrast, the 2024 resurgence showed a similar but less pronounced involvement of these age groups, suggesting ongoing but partially attenuated transmission among young adults. The persistence of cases in the same cohorts indicates that immunity gaps identified in previous outbreaks were not fully closed and may have continued to sustain viral circulation over time [23,24,25].

During the two epidemic years, the highest age-specific incidence in Tuscany was observed in individuals aged 15–24 years (22 cases; 6.75 per 100,000) and 25–44 years (59 cases; 6.89 per 100,000) in 2019, while in 2024 the highest incidence occurred among children aged 0–4 years (7 cases; 6.19 per 100,000).

This age distribution is consistent with recent national surveillance data. In Italy, 391 measles cases were notified between January and June 2025, showing a marked decline compared with the same period in 2024, although transmission persisted across all regions [26,27]. The median age of cases remained high (31 years), and nearly half occurred in individuals aged ≥15 years, indicating that measles transmission in Italy is no longer limited to childhood but increasingly involves adolescents and adults [23]. At the same time, the highest incidence rates were recorded in children aged <5 years, including infants <1 year, supporting a dual epidemiological pattern typical of settings with heterogeneous immunity profiles: sustained circulation among susceptible older age groups, with secondary transmission to unvaccinated or not yet fully protected young children [23,24,28].

In Tuscany, measles cases in 2024 were unevenly distributed across Local Health Units (LHUs), with the Tuscany North-West LHU accounting for the majority of notifications both in 2024 (*n* = 49; 65.3%) and over the 2019–2024 period (*n* = 90; 44.1%), indicating clear intra-regional clustering of transmission. A similar geographic heterogeneity was observed in neighbouring regions. In 2024, Liguria, Emilia–Romagna, and Lazio reported higher incidence rates than Tuscany (approximately 2.92, 3.16, and 3.50 cases per 100,000 population, respectively), suggesting that measles transmission was not confined to Tuscany but affected several areas of central and northern Italy. At the national level, approximately 1045 cases were reported in 2024, corresponding to an average incidence of ~1.77 cases per 100,000 population, reflecting sustained but spatially uneven viral circulation [1]. Taken together, these findings indicate that measles circulation in 2024 was characterized by considerable geographic heterogeneity at local, regional, and national levels. The concentration of cases within specific LHUs and the higher incidence observed in neighbouring regions suggest that transmission occurred in localized clusters rather than uniformly across the population. This pattern may reflect local variations in population immunity and transmission dynamics, highlighting the importance of maintaining high vaccination coverage and underscoring the need for targeted and coordinated public health interventions across regional boundaries.

International evidence supports this interpretation. Recent outbreaks in Europe and the Americas show that once vaccination coverage falls below the herd immunity threshold, measles transmission can rapidly re-establish [14,15,16]. Large outbreaks reported in several high-income countries (Romania, Belgium, the United Kingdom, the United States, Canada, and Mexico) demonstrate the vulnerability of settings where susceptible clusters persist [29,30,31]. The resurgence observed in Tuscany should therefore be interpreted not as an isolated event but as part of a wider post-elimination fragility affecting multiple countries.

The temporal dynamics observed in Tuscany must also be considered in the context of the COVID-19 pandemic. Between 2020 and 2023, non-pharmaceutical interventions such as lockdowns, reduced mobility, mask use, and school closures likely reduced measles transmission, as documented for several respiratory and contact-transmitted infections [32].

However, this temporary suppression of viral circulation occurred simultaneously with disruptions in routine immunization services and delays in vaccine uptake, leading to a progressive widening of the immunity gap—that is, the proportion of individuals lacking protective immunity in the population. In epidemiological terms, this reflects an accumulation of susceptible subjects compared with pre-pandemic years, particularly among children who missed scheduled doses and adults with incomplete vaccination histories or waning immunity [33,34].

As measles transmission resumed following the relaxation of control measures, the expanded pool of susceptible individuals likely increased the effective reproduction potential of the virus, facilitating renewed outbreaks and sustained chains of transmission. With the restoration of social mixing and contact rates, opportunities for exposure rose within a population in which susceptibility had accumulated during the pandemic period.

Similar patterns have been described for other vaccine-preventable diseases, including pertussis in Tuscany, where a marked resurgence of pertussis cases in 2024 was attributed to waning immunity combined with pandemic-related interruption of preventive services [35]. Given the high transmissibility of measles, the impact of these immunity gaps may be even greater.

The age profile observed in Tuscany deserves particular attention. The predominance of adult cases reflects a shift from the classic childhood epidemiology of measles towards a current pattern driven by incomplete immunization histories. This has important public health implications, as measles in adults is generally associated with more severe clinical outcomes and greater use of healthcare resources. At the same time, cases among infants indicate insufficient population immunity, since children under one year are not yet fully protected by routine vaccination schedules and rely on indirect protection through herd immunity and residual maternal antibodies [23,24,28].

Hospitalization data further emphasize the burden of disease. In Tuscany, more than one-third of notified cases in 2024 required hospital admission, accounting for over 40% of admissions during the study period. This proportion is epidemiologically relevant and likely reflects the older age distribution of cases, increased clinical severity in adults, precautionary admissions in vulnerable individuals, and the management of complications such as pneumonia or hepatitis. Beyond the clinical impact, measles outbreaks generate substantial pressure on health services through emergency care, inpatient management, laboratory diagnostics, case isolation, contact tracing, and infection control procedures. These costs greatly exceed those associated with preventive vaccination programmes [34].

Vaccination status remains the key determinant of measles transmission. Nationally, most cases occur among unvaccinated individuals, confirming that ongoing circulation is primarily sustained by failure to initiate or complete recommended schedules [23]. In Tuscany, the recurrence of outbreaks in 2019 and 2024 suggests persistent immunity gaps in both historical birth cohorts and in more recent generations affected by delayed uptake [21,24,25].

Although the vaccination status is not always available for individual measles cases in the surveillance systems, population-level vaccination coverage is routinely monitored in Italy and provides an important epidemiological context. According to data from the ISS and the Ministry of Health, measles-containing vaccine (MMR) coverage in Tuscany has remained consistently high and has exceeded the WHO target of 95% for herd immunity. For the 2021 birth cohort, MMR coverage at 24 months reached 97.3% in Tuscany, compared with 94.6% nationally. By 36 months of age, following catch-up vaccination activities, coverage increased to 96.3% and 95.4%, respectively. Tuscany has maintained coverage above the 95% threshold since at least the 2017 birth cohort, whereas national coverage, despite substantial improvements following the introduction of Italy’s mandatory vaccination requirements for school and preschool attendance in 2017 [9], has generally remained slightly below the WHO target at 24 months of age. These findings indicate that, overall, measles vaccine uptake in Tuscany is consistently high and exceeds the national average. Nevertheless, the occurrence of recent outbreaks highlights that transmission can still occur among susceptible individuals and within localized pockets of under-vaccination, despite high overall vaccination coverage [35].

This underscores the need to complement childhood immunization with catch-up strategies targeting adolescents and adults, vaccination of healthcare workers, systematic verification of vaccination status during healthcare encounters, and protection of women of childbearing age [20,36,37]. Although information on occupation was not always available in the analysed dataset, healthcare personnel represent a population at increased risk of exposure to measles and may contribute to healthcare-associated transmission when susceptible. This issue remains highly relevant from a public health perspective, as healthcare workers may serve as a sentinel population for identifying residual immunity gaps and may facilitate transmission to vulnerable patients in healthcare settings. These observations are consistent with international evidence indicating that healthcare workers continue to be disproportionately affected during measles outbreaks and may act as sources of transmission to susceptible patients when immunity gaps persist. Although vaccination programs for healthcare personnel are widely recommended, vaccine uptake remains suboptimal in some settings, leaving a proportion of workers susceptible to vaccine-preventable diseases. Maintaining high levels of measles immunity among healthcare personnel through occupational health surveillance, proactive vaccination strategies, and targeted educational interventions should therefore remain a priority for infection prevention and control programs [38].

This study has several limitations. First, the PREMAL surveillance system, being passive in nature, is subject to underreporting and heterogeneity in case ascertainment, which may lead to underestimation of the true measles burden. This is particularly relevant for mild, modified, or atypical presentations that are less likely to seek medical attention or be notified, whereas clinically severe cases and those requiring hospitalization are more likely to be captured. Because asymptomatic measles infection is uncommon, underascertainment is more plausibly attributable to paucisymptomatic cases, especially among partially vaccinated individuals or persons with incomplete immunity. In addition, vaccination status was not systematically recorded, precluding robust assessment of vaccine effectiveness and of the relative contribution of breakthrough versus unvaccinated cases to observed transmission dynamics. Second, vaccination data were available only for 2024, limiting the ability to explore temporal associations between changes in population immunity and measles incidence over the study period. Third, the dataset lacked detailed epidemiological, virological, and temporal information. Specifically, data on case origin (imported, import-related, or endemic), case classification (laboratory-confirmed versus epidemiologically linked), and measles virus genotypes were not available. Moreover, the absence of information on the timing of symptom onset and case notification precluded the assessment of seasonal patterns, the reconstruction of epidemic curves, and a comprehensive description of outbreak dynamics, including how the virus was introduced and subsequently spread within the community. Consequently, it was not possible to quantify the contribution of imported cases to overall transmission, characterize transmission chains, or evaluate genotype circulation in Tuscany. Fourth, the descriptive nature of the study did not allow inferential analyses to identify risk factors or assess the independent contribution of demographic, geographic, and clinical determinants of infection. In addition, given the limited number of reported cases, we did not stratify data by a single year of age within the 0–4-year age group. As a result, it was not possible to distinguish cases occurring among infants younger than 12 months, who were not yet eligible for routine measles vaccination, from those occurring among vaccine-eligible children aged 1–4 years. Overall, our findings illustrate how measles can rapidly reemerge when immunity gaps persist, even in settings with longstanding vaccination programmes. High-quality surveillance remains essential for the timely detection of outbreaks, monitoring of transmission patterns, identification of vulnerable populations, assessment of disease severity, and implementation of appropriate public health responses. Future studies integrating routine epidemiological surveillance with molecular surveillance data and more granular temporal information would provide a more comprehensive understanding of measles transmission dynamics and support more targeted prevention and control strategies. In the current post-pandemic context, strengthening surveillance alongside proactive vaccination strategies will be crucial to prevent future outbreaks and advance progress toward sustainable measles elimination.

## 5. Conclusions

In Tuscany, vaccination strategies must go beyond paediatrics, implementing targeted vaccination campaigns for young adults, actively involving general practitioners in assessing immunization status, and offering vaccinations at other times, such as screening programs, antenatal care, travel health centres, or preventive medicine visits. At the same time, strengthening surveillance systems, international collaboration, and ensuring timely outbreak investigations remain essential to achieving the measles elimination targets established by the WHO.

## Data Availability

Data were obtained from the national surveillance system (PREMAL).

## Supplementary Materials

The following supporting information can be downloaded at: https://www.mdpi.com/article/10.3390/vaccines14070558/s1, Table S1: Summary of data presented in the article “Measles surveillance in Tuscany, Italy: a six-year epidemiological analysis (2019–2024)”.

## Abbreviations

The following abbreviations are used in this manuscript:

PREMAL	Sistema per la sorveglianza delle malattie infettive (national surveillance system).
LHU	Local Health Unit
ISTAT	Istituto Nazionale di Statistica (National Institute of Statistics)
ISS	Istituto Superiore di Sanità (National Health Institute)
ECDC	European Centre for Disease Prevention and Control
