# Supplementary material for: Measles Surveillance in Tuscany (Italy), 2019–2024: A Six-Year Epidemiological Analysis
Source: Vaccines (Basel). 2026 Jun 25;14(7):558. doi: 10.3390/vaccines14070558 (PMC13417429; doi:10.3390/vaccines14070558)
Supplement: Supplementary file 1 [file vaccines-14-00558-s001.zip › vaccines-4357279-supplementary.pdf]

Supplementary Table S1:

Summary of data presented in the article “Measles surveillance in Tuscany, Italy: a six-year epidemiological analysis (2019–2024)”

For the purpose of completing the table, incidences are reported with two decimal places and percentages with one decimal place, both rounded. Cumulative incidence across multiple years of analysis were calculated by using the average resident population over the observation period as denominator.

Population (A): Number of measles Cases and Incidence Rates (*cases/100,000)* in Tuscany (Italy), 2019–2024

Hospitalizations (B): Number of measles hospitalized Cases and Hospitalisation Rates (*cases/100,000)* in Tuscany (Italy), 2019–2024

**Population (A)**		**2019**	**2020**	**2021**	**2022**	**2023**	**2024**	**2019-2024**	**Mean annual incidence rate (19-24)**
**General population (M+F)**	**N. of cases**	116	7		2	4	75	204	
	Incidence	3.13	0,19		0.05	0.03	2.05	5.58	0.93
	C.I.	(2.56-3.71)	(0.05-0.33)		(0.01-0.20)	(0.03-0.28)	(1.59-2.51)	(4.78-6.31)	(0.80-1.07)
**Male**	**N. of cases**	45	2	-	1	2	25	75	
	Incidence	2.52	0.11		0.06	0.11	1.40	4.21	0.70
	C.I	(1.78-3.25)	(0.01-0.40)		(0.001-0.31)	(0.01-0.41)	(0.85-1.95)	(3.26-5.16)	(0.54-0.86)
**Female**	N. of cases	71	5		1	2	50	129	
	Incidence	3.71	0.26		0.05	0.11	2.70	6.80	1.13
	C.I.	(2.84-4.58)	(0.09-0.61)		(0.001-0.30)	(0.01-0.38)	(1.92-3.39)	(5.63-7.98)	(0.94-1.33)
**0-4 years**	N. of cases	6	1		-	1	7	15	
	Incidence	4.48	0.78			0.86	6.19	12.19	2.03
	C.I.	(0.74-8.22)	(0.02-4.33)			(0.02-4.78)	(1.79-10.59)	(6.02-18.36)	(1.00-3.06)
**5-14 years**	N. of cases						7	7	
	Incidence						2.33	2.23	0.37
	C.I.						(0.64-4.01)	(0.58-3.88)	(0.10-0.65)
**15-24 years**	N. of cases	22	3		-		9	34	
	Incidence	6.75	0.91				2.62	10.17	1.70
	C.I.	(3.97-9.54)	(0.19-2.67)				(0.88-4.35)	(6.75-13.59)	(1.13-2.27)
**25-44 years**	N. of cases	59	2			2	31	94	
	Incidence	6.89	0.24			0.25	3.96	11.56	1.93
	C.I.	(5.09-8.70)	(0.03-0.87)			0.03-0.91)	(2.59-5.33)	(9.22-13.89)	(1.54-2.32)

**45-64 years**	N. of cases	26	1	2	1	19	49	
	Incidence	2.32	0.09	0.17	0.09	1.65	4.30	0.72
	C.I.	(1.44-3.21)	(0.00-0.49)	(0.02-0.63)	(0.00-0.48)	(0.90-2.40)	(3.10-5.51)	(0.52-0.92)
**≥65 years**	N. of cases	3				2	5	
	Incidence	0.32				0.21	0.52	0.09
	C.I.	(0.07-0.93)				(0.03-0.75)	(0.17-1.22)	0.03-0.20)
**LHU Central Tuscany**	N. of cases	45	7	2	1	18	73	
	Incidence	2.79	0.43	0.12	0.06	1.12	4.54	0.76
	C.I.	(1.97-3.60)	(0.11-0.76)	(0.02-0.45)	(0.002-0.35)	(0.60-1.64)	(3.50-5.58)	(0.58-0.93)
**LHU North-West**	N. of cases	40	-	-	1	49	90	
	Incidence	3.15			0.08	3.93	7.18	1.20
	C.I.	(2.17-4.13)			(0.002-0.45)	(2.84-5.03)	(5.70-8.66)	(0.95-1.44)
**LHU South-East**	N. of cases	31			2	8	41	
	Incidence	3.73			0.25	0.99	5.01	0.84
	C.I.	(2.40-5.05)			(0.03-0.89)	(0.43-1.95)	(3.48-6.55)	(0.58-1.09)

Hospitalization (B)

**Total**	N. of hospitalization cases	52	4	-	3	26	85	
	% hospitalization/ total of cases of the year	44.8%	57.1%		75.0%	34.7%	41.7%	
	Hospitalization Rates	1.42	0.11		0.08	0.71	2.32	0.38
	C.I.	(1.03-1.80)	(0.03-0.28)		(0.02-0.24)	(0.44-0.98)	(1.82-2.80)	
